# Inter-species horizontal transfer resulting in core-genome and niche-adaptive variation within *Helicobacter pylori*

**DOI:** 10.1186/1471-2164-6-9

**Published:** 2005-01-27

**Authors:** Nigel J Saunders, Prawit Boonmee, John F Peden, Stephen A Jarvis

**Affiliations:** 1 Bacterial Pathogenesis and Functional Genomics Group, The Sir William Dunn School of Pathology, University of Oxford, South Parks Road, Oxford, OX1 3RE, UK; 2 Department of Computer Science, University of Warwick, Coventry, CV4 7AL,UK; 3 Oxford University Bioinformatics Centre, Sir William Dunn School of Pathology, University of Oxford, South Parks Road, Oxford, OX1 3RE, UK

## Abstract

**Background:**

Horizontal gene transfer is central to evolution in most bacterial species. The detection of exchanged regions is often based upon analysis of compositional characteristics and their comparison to the organism as a whole. In this study we describe a new methodology combining aspects of established signature analysis with textual analysis approaches. This approach has been used to analyze the two available genome sequences of *H. pylori*.

**Results:**

This gene-by-gene analysis reveals a wide range of genes related to both virulence behaviour and the strain differences that have been relatively recently acquired from other sequence backgrounds. These frequently involve single genes or small numbers of genes that are not associated with transposases or bacteriophage genes, nor with inverted repeats typically used as markers for horizontal transfer. In addition, clear examples of horizontal exchange in genes associated with 'core' metabolic functions were identified, supported by differences between the sequenced strains, including: *ftsK*, *xerD *and *polA*. In some cases it was possible to determine which strain represented the 'parent' and 'altered' states for insertion-deletion events. Different signature component lengths showed different sensitivities for the detection of some horizontally transferred genes, which may reflect different amelioration rates of sequence components.

**Conclusion:**

New implementations of signature analysis that can be applied on a gene-by-gene basis for the identification of horizontally acquired sequences are described. These findings highlight the central role of the availability of homologous substrates in evolution mediated by horizontal exchange, and suggest that some components of the supposedly stable 'core genome' may actually be favoured targets for integration of foreign sequences because of their degree of conservation.

## Background

*Helicobacter pylori *is a bacterial pathogen associated with gastritis, peptic ulcers, gastric adenocarcinoma, and rare lymphomas [1]. It has a highly panmictic population structure in which homologous recombination makes the predominant contribution to sequence differences within a highly diverse population structure [2]. The acquisition of genes from other strains and species is by far the most rapid evolutionary process. This occurs frequently without loss of existing functions, is central to the evolution of niche-adaptive and pathogenic characteristics of bacteria, and greatly influences inter-strain differences in gene complement [3-5]. In this context, it is notable that none of the traits typically used to differentiate *E. coli *from *Salmonella *can be attributed to point mutation genes but are broadly attributable to horizontal exchange [6]. *H. pylori *is relatively unusual in that it is a naturally transformable Gram-negative species that does not appear to have a species-specific DNA uptake sequence and appears to rely upon its niche separation as a transformation barrier [7]. Disease associated *H. pylori *strains have been divided into two types, type I being those that carry the *cag *pathogenicity island [8] (*cag *PAI), which has a foreign species origin, and are associated with more severe disease.

Dinucleotide composition is highly stable within a genome and can distinguish between sequences from different species. Based upon its constancy the species composition is referred to as a 'genome signature' [9,10]. This characteristic has been applied to assessments of DNA metabolic processes such as methylation and base conversion, DNA structure, and evolutionary relationships. It has also become established as a method for the identification of sequences that have been acquired by inter-species horizontal transfer. For example, lateral transfer has recently been shown using these methods for a tryptophan pathway operon [11], the gain of additional metabolic functions in *Pseudomonas putida *[12], a determination that many gain of function genes have been acquired by *E. coli *rather than lost from *S. typhi *[13], and more recently developed Bayesian methods based upon similar premises have been used to assess global signatures and determine the origins of some lateral transfer events [14,15]. However there are problems associated with this and other methods that use progressive 'walking windows', and the larger the window the greater the problems. These result from the inclusion of intergenic sequence, the inability to distinguish divergences due to a single highly divergent gene from that from a cluster of less divergent ones, and an inability to identify the limits of the abnormal regions. In practice additional features are necessary to determine the ends of such regions, such as the location of repeats typical of pathogenicity islands in *H. pylori *[16], or comparisons with other sequences as in *N. meningitidis *strain MC58 [17]. In addition, divergence scores are influenced by the size of the sampling window used such that sampling effects limit analysis of sequences shorter than about 800 bp (data not presented), and the need to use fixed window sizes prevents gene by gene studies.

We describe the use of a linear implementation of signature analysis that can efficiently address a range of walking window sizes using dinucleotide signatures (DNS) and longer signatures. In addition, use of a new approach based upon classical text analysis that allows analysis of genomes gene-by-gene is described. Analysis of *H. pylori *sequences, combined with comparisons of the identified genes between genomes, reveals complex changes that influence both niche-adaptive and core functions illustrating a previously unpredicted range of functions which are continuously undergoing variation and selection.

## Results and discussion

**Figure 1:**
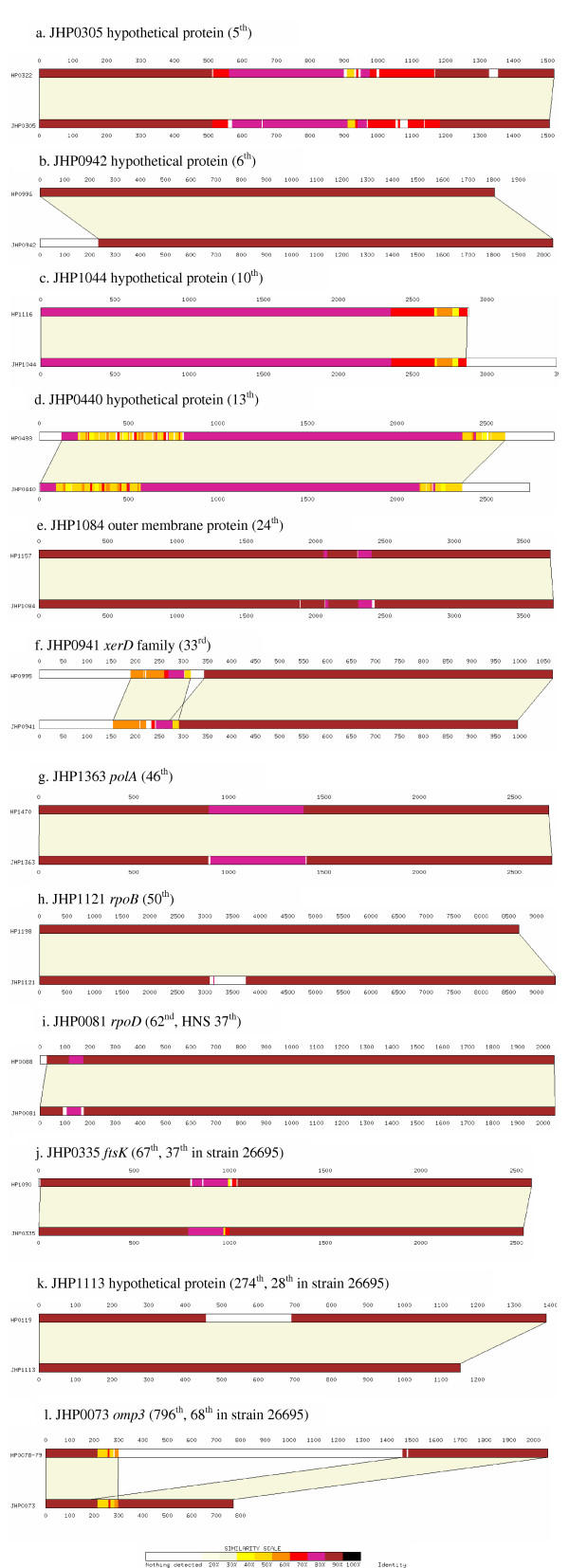
Comparisons using LAlign between a representative selection of orthologous genes with divergent DNA present in both *H. pylori *strains J99 and 26695 (presented in descending order of divergence as determined in strain J99).

Genes were ranked on the basis of their divergence from the mean genome composition. The degree of divergence that is indicative of acquisition from other species is not an absolute. The frequency with which genes are acquired, the untypicality of the donated material, and the rate at which they are ameliorated to the host sequence composition influence it. Strains J99 and 26695 had 53 (Table 1) and 60 (Table 4) genes respectively with DNS that were >2 SD from the mean. Those with annotated functions included genes from the *cag *pathogenicity island (6 and 5), *vac *and related toxins (3 and 4), and restriction-modification genes (2 and 4). On the basis of the similarities determined in the *H*. *pylori *strain J99 sequence annotation, 7 of the most divergent genes as determined by DNS are not present in strain 26695. Likewise, 2 of the 50 most divergent genes in strain 26695 are not present in strain J99. This is consistent with the identification of genes acquired from other species that have not extended to both sequenced strains. It also suggests that a significant proportion of the 6 to 7% of genes unique to one or other strain [18] are inherent to the *Helicobacter *gene pool, but are variably present in different strains rather than reflecting recent foreign origins. Comparisons of a selection of identified orthologous genes in the two strains are shown in Figure 1.

**Table 1:** The 53 most divergent (>2 SD) genes in H. pylori strain J99 by DNS showing their ranking in strain 26695 and in TNS and HNS analysis

**DNS order**	**JHP #**	**annotation**	**26695 #**	**26695 DNS order**	**TNS order**	**HNS order**
**1**	JHP0952	hypothetical protein	HP0427	**14**	**3**	1355
**2**	JHP0476	cag pathogenicity island protein (cag7)	HP0527	**1**	**2**	**2**
**3**	JHP0556	vacuolating cytotoxin (vacA) paralog	HP0609/10	**4/13**	**5**	**4**
**4**	JHP0274	vacuolating cytotoxin (vacA) paralog	HP0289	**2**	**6**	**5**
**5**	JHP0305	hypothetical protein	HP0322	**3**	**8**	**10**
**6**	JHP0942	hypothetical protein	HP0996	**5**	**13**	27
**7**	JHP0856	vacuolating cytotoxin (vacA) paralog	HP0922	**6**	**9**	**6**
**8**	JHP0050	hypothetical protein	HP0058	88	**7**	84
**9**	JHP1300	hypothetical protein	HP1408	**15**	**1**	**1**
**10**	JHP1044	hypothetical protein	HP1116	**8**	**14**	**8**
**11**	JHP0928	hypothetical protein	NAH	-	**12**	**9**
**12**	JHP0074	hypothetical protein	HP0080	**9**	**32**	125
**13**	JHP0440	hypothetical protein	HP0488	**7**	**16**	**17**
**14**	JHP1042	hypothetical protein	HP1115	**20**	**25**	694
**15**	JHP1321	histidine and glutamine-rich metal-binding protein	HP1432	**46**	**4**	49
**16**	JHP0934	hypothetical protein	NAH	-	**15**	95
**17**	JHP0495	cag island protein (cagA)	HP0547	**31**	**20**	**12**
**18**	JHP0931	topoisomerase I (topA 3)	NAH	-	**18**	**20**
**19**	JHP0693	hypothetical protein	HP0756	**24**	59	1490
**20**	JHP0632	N-methylhydantoinase	HP0696	**19**	**44**	36
**21**	JHP0471	cag pathogenicity island protein (cag3)	HP0522	**11**	**35**	62
**22**	JHP0438	outer membrane protein	HP0486	**26**	67	145
**23**	JHP0026	hypothetical protein	HP0030	**45**	**36**	64
**24**	JHP1084	outer membrane protein (omp26)	HP1157	34	**17**	24
**25**	JHP0481	cag island protein (cagT)	HP0532	**23**	70	558
**26**	JHP0052	hypothetical protein	HP0059	**43**	**24**	120
**27**	JHP0336	hypothetical protein	HP1089	**12**	51	54
**28**	JHP1426	iron(III) dicitrate transport protein (fecA)	HP1400	**32**	78	111
**29**	JHP0174	hypothetical protein	HP0187 / 8 / 6	47&1127&596	88	90
**30**	JHP1297	type III restriction enzyme (res)	NAH	-	63	28
**31**	JHP0953	hypothetical protein	NAH	-	**26**	1463
**32**	JHP0067	urease beta subunit (urea amidohydrolase) (ureB)	HP0072	**21**	**37**	70
**33**	JHP0941	integrase/recombinase (xerD)	HP0995	**25**	100	541
**34**	JHP0548	flagellin A (flaA)	HP0601	**33**	**40**	154
**35**	JHP0299	hypothetical protein	HP061/2	230&765	**11**	275
**36**	JHP1033	hypothetical protein	HP1106	**59**	262	342
**37**	JHP1409	type II restriction enzyme (methyltransferase)	NAH	-	55	**15**
**38**	JHP0626	iron(III) dicitrate transport protein (fecA)	HP0686	62	89	47
**39**	JHP0940	hypothetical protein	NAH	-	53	393
**40**	JHP1253	hypothetical protein	HP1333	**40**	75	384
**41**	JHP0132	cytochrome oxidase (cbb3 type) (fixN)	HP0144	**27**	206	209
**42**	JHP0842	hypothetical protein	HP0906	**42**	**29**	**21**
**43**	JHP0925	hypothetical protein	NAH	-	130	990
**44**	JHP0613	hypothetical protein	HP0669	69	**42**	33
**45**	JHP0565	DNA mismatch repair protein (mutS)	HP0621	**22**	227	82
**46**	JHP1363	DNA polymerase I (polA)	HP1470	**30**	81	46
**47**	JHP0489	cag island protein (cagH)	HP0541	71	137	398
**48**	JHP1260	siderophore-mediated iron transport protein (tonB)	HP1341	85	1260	402
**49**	JHP0492	DNA transfer protein (cagE)	HP0544	104	95	50
**50**	JHP1121	DNA-directed RNA polymerase, beta subunit (rpoB)	HP1198	84	**23**	**16**
**51**	JHP1434	DNA repair protein (recN)	HP1393	**35**	177	160
**52**	JHP0491	cag island protein (cagF)	HP0543	82	170	828
**53**	JHP0191	hypothetical protein	HP0205	**57**	**33**	**7**

Genes with > 2 SD divergence indicated in **bold**

NAH indicates No Annotated Homologue in the other sequence

**Table 4:** Top 60 most divergent (>2 SD) genes by DNS in *H. pylori *strain 26695 plus those additional genes in the top 50 genes from TNS and HNS

**DNS order**	**annotation**	**HP#**	**J99 #**	**J99 DNS order**	**TNS order**	**HNS order**
**1**	cag pathogenicity island protein (cag7)	HP0527	JHP0476	**2**	**1**	**1**
**2**	vacuolating cytotoxin (vacA) paralog	HP0289	JHP0274	**4**	**2**	**4**
**3**	poly E-rich hypothetical protein	HP0322	JHP0305	**5**	**8**	**5**
**4**	hypothetical protein	HP0609	JHP0556*	**3**	**6**	**9**
**5**	hypothetical protein	HP0996	JHP0942	**6**	**14**	46
**6**	vacuolating cytotoxin (vacA) paralog	HP0922	JHP0856	**7**	**5**	**3**
**7**	hypothetical protein	HP0488	JHP0440	**13**	**10**	**12**
**8**	hypothetical protein	HP1116	JHP1044	**10**	**11**	**13**
**9**	hypothetical protein	HP0080	JHP0074	**12**	**18**	122
**10**	hypothetical protein	HP0489	JHP0441	115	**36**	582
**11**	cag pathogenicity island protein (cag3)	HP0522	JHP0471	**21**	**48**	100
**12**	hypothetical protein	HP1089	JHP0336	**27**	67	59
**13**	vacuolating cytotoxin (vacA) paralog	HP0610	JHP0556*	**3**	**12**	**17**
**14**	hypothetical protein	HP0427	JHP0952	**1**	**3**	737
**15**	hypothetical protein	HP1408	JHP1300	**9**	**4**	738
**16**	type III restriction enzyme R protein (res)	HP0592	NAH	-	**30**	35
**17**	hypothetical protein	HP0119	NAH	-	**7**	**2**
**18**	vacuolating cytotoxin (vacA)	HP0887	JHP0819	59	**25**	34
**19**	N-methylhydantoinase	HP0696	JHP0632	**20**	**35**	43
**20**	hypothetical protein	HP1115	JHP1042	**14**	**33**	866
**21**	urease beta subunit (urea amidohydrolase) (ureB)	HP0072	JHP0067	**32**	**38**	87
**22**	DNA mismatch repair protein (MutS)	HP0621	JHP0565	**45**	137	64
**23**	cag island protein (cagT)	HP0532	JHP0481	**25**	87	693
**24**	hypothetical protein	HP0756	JHP0693	**19**	71	1548
**25**	integrase/recombinase (xerD)	HP0995	JHP0941	**33**	**39**	448
**26**	outer membrane protein	HP0486	JHP0438	**22**	147	142
**27**	cytochrome oxidase (cbb3 type) (fixN)	HP0144	JHP0132	**41**	102	168
**28**	type IIS restriction enzyme R and M protein (ECO57IR)	HP1517	NAH	-	**42**	**14**
**29**	DNA transfer protein (cagE)	HP0441	JHP0492	**49**	**51**	22
**30**	DNA polymerase I (polA)	HP1470	JHP1363	**46**	77	54
**31**	cag island protein (cagA)	HP0547	JHP0495	**17**	**15**	**7**
**32**	iron(III) dicitrate transport protein (fecA)	HP1400	JHP1426	**28**	99	129
**33**	flagellin A (flaA)	HP0601	JHP0548	**34**	**40**	180
**34**	outer membrane protein (omp26)	HP1157	JHP1084	**24**	**17**	25
**35**	DNA repair protein (recN)	HP1393	JHP1434	**51**	154	207
**36**	type I restriction enzyme R protein (hsdR)	HP0464	NAH	-	90	26
**37**	cell division protein (ftsK)	HP1090	JHP0335	67	181	90
**38**	hypothetical protein	HP1003	NAH	-	61	170
**39**	histidine-rich, metal binding polypeptide (hpn)	HP1427	NAH	-	**26**	1449
**40**	hypothetical protein	HP1333	JHP1253	**40**	**53**	296
**41**	hypothetical protein	HP0788	JHP0725	68	72	256
**42**	hypothetical protein	HP0906	JHP0842	**42**	**22**	**16**
**43**	hypothetical protein	HP0059	JHP0052	**26**	**21**	320
**44**	GMP reductase (guaC)	HP0854	JHP0790	107	169	451
**45**	hypothetical protein	HP0030	JHP0026	**23**	**24**	39
**46**	histidine and glutamine-rich metal-binding protein	HP1432	JHP1321	**15**	**9**	1432
**47**	hypothetical protein	HP0186	JHP0174	**29**	130	276
**48**	fucosyltransferase	HP0651	JHP0596	105	**43**	75
**49**	translation elongation factor EF-Tu (tufB)	HP1205	JHP1128	81	64	166
**50**	virulence associated protein homolog (vacB)	HP1248	JHP1169	79	164	160
**51**	hypothetical protein	HP0449	NAH	-	81	449
**52**	type III restriction enzyme R protein	HP1371	JHP1285	55	119	23
**53**	virB4 homolog (virB4)	HP0459	NAH	-	**49**	28
**54**	2',3'-cyclic-nucleotide 2'-phosphodiesterase (cpdB)	HP0104	JHP0096	56	73	68
**55**	hypothetical protein	HP1479	JHP1372	135	153	127
**56**	RNA polymerase sigma-70 factor (rpoD)	HP0088	JHP0081	62	**55**	31
**57**	hypothetical protein	HP0205	JHP0191	**53**	78	**8**
**58**	hypothetical protein	HP1143	JHP1071	78	**29**	41
**59**	hypothetical protein	HP1106	JHP1033	**36**	272	277
**60**	cag pathogenicity island protein (cag13)	HP0534	JHP0482	71	225	1021
						
63	DNA topoisomerase I (topA)	HP0440	NAH	-	149	24
68	outer membrane protein (omp3)	HP0079	JHP0073	796	**45**	99
69	hypothetical protein	HP0669	JHP0613	**44**	60	42
74	cag pathogenicity island protein (cag8)	HP0528	JHP0477	72	**50**	27
75	hypothetical protein	HP0453	NAH	-	58	**10**
84	DNA-directed RNA polymerase, beta subunit (rpoB)	JHP1121	**50**	**23**	19	
91	hypothetical protein	HP1142	JHP1070	60	**19**	**6**
97	multidrug resistance protein (spaB)	HP0600	JHP0547	75	**41**	30
103	type I restriction enzyme R protein (hsdR)	HP1402	JHP1424	195	86	21
109	adenine/cytosine DNA methyltransferase	HP0054	NAH	-	120	20
119	preprotein translocase subunit (secA)	HP0786	JHP0723	159	176	49
121	hypothetical protein	HP0058	JHP0051	394	**16**	53
122	hypothetical protein	HP0513	JHP0462	104	**28**	**15**
125	type I restriction enzyme M protein (hsdM)	HP1403	JHP1423	299	340	44
132	hypothetical protein	HP0731	JHP0668	110	80	32
139	hypothetical protein	HP0508	JHP0458	84	**32**	77
142	hypothetical protein	HP1187	JHP1113	274	**31**	38
167	hypothetical protein	HP1520	NAH	-	**20**	33
179	hypothetical protein	HP0118	JHP0110	64	**27**	36
195	type III restriction enzyme R protein (res)	HP1521	JHP1410	161	210	18
209	outer membrane protein (omp17)	HP0725	JHP0662	257	**47**	101
224	hypothetical protein	HP0733	JHP0670	769	222	48
230	hypothetical protein	HP0611	JHP0299	**35**	**37**	1129
249	hypothetical protein	HP0345	NAH	-	**46**	1338
283	hypothetical protein	HP0120	NAH	-	**44**	50
291	translation initiation factor IF-2 (infB)	HP1048	JHP0377	330	332	45
297	DNA polymerase III alpha-subunit (dnaE)	HP1460	JHP1353	509	219	47
342	type I restriction enzyme R protein (hsdR)	HP0846	JHP0784	244	101	37
363	adenine specific DNA methyltransferase (mod)	HP1522	JHP1411	857	207	**11**
410	secreted protein involved in flagellar motility	HP1192	JHP1117	614	**13**	1256
593	hypothetical protein	HP1516	NAH	-	**34**	1090
631	hypothetical protein	HP0586	JHP0534	577	163	29
1080	type II restriction enzyme (methyltransferase)	HP0478	JHP0430	953	220	40

* probably frame shifted components of the same *vacA *related gene

Genes with > 2 SD divergence in each analysis are indicated in **bold**

NAH indicates No Annotated Homologue in the other sequence

It cannot be assumed that all genes identified in this manner have been recently acquired. It is necessary to assess the nature of the sequence to determine if its divergence might be accounted for on the basis of features of the encoded protein. For example, JHP0476/HP0527, JHP1300/HP1408 and JHP0074/HP0080 include repetitive sequences likely to account for their DNS divergence. This type of analysis cannot be used to determine the possible foreign origin of such genes. Notably, the most divergent *cag *PAI gene (the 1^st ^and 2^nd ^most divergent gene in the whole genomes of strain 26695 and J99 respectively, JHP0476/HP0527) has a highly complex repetitive structure and the size of the large divergent peak associated with this island using previous methods is largely due to the presence of this gene.

While a significant proportion of the genes identified in this analysis are associated with regions including several such genes and which share characteristics of islands of horizontal transfer or pathogenicity islands, this is far from universally true. There are many instances of single genes or small numbers of genes that are present that are not associated with any features that might otherwise have been used as indicators of horizontal acquisition such as transposases and flanking repeats.

Our initial goal was to identify recently acquired and exchanged genes as candidates likely to be important in niche-adaptation, host interactions, and alterations in bacterial fitness. It has been argued that essential genes are unlikely to be transferred successfully since recipient taxa would already bear functional orthologues, which would have experienced long-term co-evolution with the rest of the cellular machinery. In contrast, it is proposed that those under weak or transient selection – like those associated with nonessential catabolic processes, new operons, and those providing new niche-adaptive changes are likely to be successfully transferred and retained [19]. This leads to a model in which a stable 'core genome' comprised of essential metabolic, regulatory, and cell division genes provides a stable context for the more labile non-essential and niche adaptive genes. On this basis such genes are used for phylogenetic studies and are thought to provide a relatively constant background in which species evolution occurs. Many of the genes identified for which functions are known affect virulence or niche adaptive genes, including: the vacuolating cytotoxin and related toxins (2 and 3), urease and flagellar components, and genes involved in iron acquisition. However, we also find clear evidence, confirmed by differences between the two genome sequences, that recent, and therefore relatively frequent, horizontal transfer is not limited to genes associated with niche adaptation and virulence. Amongst the core function genes identified were *mut*S, *fts*K, *xer*D, and *pol*A. The comparisons of the latter three between the sequence strains are shown in Figure 1f,g &1j. These comparisons support the results suggesting that these genes have been the substrates for horizontal exchange between species.

Tetranucleotide composition has been used for the consideration of the presence of palindromic sequences that might be substrates for restriction systems and Chi sites and the presence of unstable repeats mediating phase variation [10], but the use of longer component signatures has not been used to identify horizontally acquired regions in bacterial genomes. Following analysis of eukaryotic sequences it was concluded that DNS captures most of the departure from randomness in DNA sequences and that longer component lengths correlate highly with the DNS results [20]. Also, analysis of dinucleotides separated by no, one, or two other nucleotides showed that separated pairs are more nearly random than adjacent pairs and were concluded to be relatively uninformative [9]. However, in preliminary analyses, while results using the typically long walking windows gave concordant results as previously reported, we found that the use of smaller walking windows generated progressively more different patterns of divergence with other length components. Using tetranucleotide (TNS) and hexanucleotide (HNS) signature analysis we find that, while in some instances there is significant overlap between the genes identified using the different component lengths, there are substantial differences that indicate additional horizontally transferred genes not identified by DNS alone (Tables 2 to 6).

**Table 2:** Top 50 most divergent genes by TNS in *H. pylori *strain J99 plus those additional genes > 2 SD greater than the mean by DNS and the 50 most divergent by HNS

**TNS order**	**Annotation**	**JHP #**	**26695 #**	**DNS order**	**HNS order**
**1**	hypothetical protein	JHP1300	HP1408	**9**	**1**
**2**	cag pathogenicity island protein (cag7)	JHP0476	HP0527	**2**	**2**
**3**	hypothetical protein	JHP0952	HP0427	**1**	1355
**4**	histidine and glutamine-rich metal-binding protein	JHP1321	HP1432	**15**	49
**5**	vacuolating cytotoxin (vacA) paralog	JHP0556	HP0609/10	**3**	**4**
**6**	vacuolating cytotoxin (vacA) paralog	JHP0274	HP0289	**4**	**5**
**7**	hypothetical protein	JHP0050	HP0058	**8**	84
**8**	hypothetical protein	JHP0305	HP0322	**5**	**10**
**9**	vacuolating cytotoxin (vacA) paralog	JHP0856	HP0922	**7**	**6**
**10**	type I restriction enzyme (hsdS)	JHP1422	NAH	319	**3**
**11**	hypothetical protein	JHP0299	HP061/2	**35**	275
**12**	hypothetical protein	JHP0928	NAH	**11**	**9**
**13**	hypothetical protein	JHP0942	HP0996	**6**	27
**14**	hypothetical protein	JHP1044	HP1116	**10**	**8**
**15**	hypothetical protein	JHP0934	NAH	**16**	95
**16**	hypothetical protein	JHP0440	HP0488	**13**	**17**
**17**	outer membrane protein (omp26)	JHP1084	HP1157	**24**	24
**18**	topoisomerase I (topA 3)	JHP0931	NAH	**18**	**20**
**19**	hypothetical protein	JHP0318	NAH	286	293
**20**	cag island protein (cagA)	JHP0495	HP0547	**17**	**12**
**21**	hypothetical protein	JHP0110	HP0118	64	**19**
**22**	hypothetical protein	JHP1208	HP1288	91	830
**23**	DNA-directed RNA polymerase, beta subunit (rpoB)	JHP1121	HP1198	**50**	**16**
**24**	hypothetical protein	JHP0052	HP0059	**26**	120
**25**	hypothetical protein	JHP1042	HP1115	**14**	694
**26**	hypothetical protein	JHP0953	NAH	**31**	1463
**27**	hypothetical protein	JHP1070	HP1142	60	**14**
**28**	hypothetical protein	JHP1113	HP1187	274	39
**29**	hypothetical protein	JHP0842	HP0906	**42**	**21**
**30**	type II restriction enzyme	JHP0630	NAH	173	588
**31**	histidine-rich, metal binding polypeptide (hpn)	JHP1320	HP1427	70	1404
**32**	hypothetical protein	JHP0074	HP0080	**12**	125
**33**	hypothetical protein	JHP0191	HP0205	**53**	**7**
**34**	hypothetical protein	JHP0376	HP1049	235	1128
**35**	cag pathogenicity island protein (cag3)	JHP0471	HP0522	**21**	62
**36**	hypothetical protein	JHP0026	HP0030	**23**	64
**37**	urease beta subunit (urea amidohydrolase) (ureB)	JHP0067	HP0072	**32**	70
**38**	hypothetical protein	JHP0939	HP0991	116	156
**39**	multidrug resistance protein (spaB)	JHP0547	HP0600	75	**18**
**40**	flagellin A (flaA)	JHP0548	HP0601	**34**	154
**41**	hypothetical protein	JHP1071	HP1143	78	61
**42**	hypothetical protein	JHP0613	HP0669	**44**	33
**43**	hypothetical protein	JHP0623	HP0682	231	1186
**44**	N-methylhydantoinase	JHP0632	HP0696	**20**	36
**45**	hypothetical protein	JHP1049	NAH	278	470
46	vacuolating cytotoxin (vacA)	JHP0819	HP0887	59	38
47	putative restriction enzyme	JHP0164	NAH	88	43
48	type I restriction enzyme R protein (hsdR)	JHP0784	HP0846	244	35
49	hook assembly protein, flagella (flgD)	JHP0843	HP0907	103	175
50	hypothetical protein	JHP0458	HP0508	84	44
					
51	hypothetical protein	JHP0336	HP1089	**27**	54
53	hypothetical protein	JHP0940	NAH	**39**	393
54	hypothetical protein	JHP0462	HP0513	104	**11**
55	type II restriction enzyme (methyltransferase)	JHP1409	NAH	**37**	**15**
58	hypothetical protein	JHP1285	HP1371	55	25
59	hypothetical protein	JHP0693	HP0756	**19**	1490
62	cag pathogenicity island protein (cag8)	JHP0477	HP0528	72	31
63	type III restriction enzyme (res)	JHP1297	NAH	**30**	28
64	hypothetical protein	JHP0668	HP0731	110	32
67	outer membrane protein	JHP0438	HP0486	**22**	145
70	cag island protein (cagT)	JHP0481	HP0532	**25**	558
71	RNA polymerase sigma-70 factor (rpoD)	JHP0081	HP0088	62	37
75	hypothetical protein	JHP1253	HP1333	**40**	384
78	iron(III) dicitrate transport protein (fecA)	JHP1426	HP1400	**28**	111
81	DNA polymerase I (polA)	JHP1363	HP1470	**46**	46
85	type I restriction enzyme (hsdS)	JHP0414	NAH	275	30
88	hypothetical protein	JHP0174	HP0187/8/6	**29**	90
89	iron(III) dicitrate transport protein (fecA)	JHP0626	HP0686	**38**	47
95	DNA transfer protein (cagE)	JHP0492	HP0544	**49**	50
100	integrase/recombinase (xerD)	JHP0941	HP0995	**33**	541
104	type III restriciton enzyme (mod)	JHP1411	HP1522	857	**13**
105	type I restriction enzyme R protein (hsdR)	JHP0416	HP0464	63	29
122	adenine specific DNA methyltransferase (mod)	JHP0244	HP0260	236	48
130	hypothetical protein	JHP0925	NAH	**43**	990
137	cag island protein (cagH)	JHP0489	HP0541	**47**	398
138	type I restriction enzyme (hsdR)	JHP1424	HP1402	195	**22**
158	hypothetical protein	JHP0540	NAH	674	26
170	cag island protein (cagF)	JHP0491	HP0543	**52**	828
177	DNA repair protein (recN)	JHP1434	HP1393	**51**	160
190	type III restriction enzyme (mod)	JHP1296	NAH	121	34
196	role in outermembrane permeability (imp)	JHP1138	HP1215/6	208	45
206	cytochrome oxidase (cbb3 type) (fixN)	JHP0132	HP0144	**41**	209
227	DNA mismatch repair protein (mutS)	JHP0565	HP0621	**45**	82
230	hypothetical protein	JHP0534	HP0586	577	40
258	type III restriction enzyme (res)	JHP1410	HP1521	161	23
262	hypothetical protein	JHP1033	HP1106	**36**	342
281	translation initiation factor IF-2 (infB)	JHP0377	HP1048	330	42
290	type II restriction enzyme (methyltrasferase)	JHP1284	NAH	750	41
1260	siderophore-mediated iron transport protein (tonB)	JHP1260	HP1341	**48**	402

Genes with > 2 SD divergence in each analysis are indicated in **bold**

NAH indicates No Annotated Homologue in the other sequence

**Table 3:** Top 50 most divergent genes by HNS in *H. pylori *strain J99 plus those additional genes >2 SD greater than the mean by DNS and top 50 by TNS

**HNS order**	**J99 annotation**	**JHP #**	**26695 #**	**DNS order**	**TNS order**
**1**	hypothetical protein	JHP1300	HP1408	**9**	**1**
**2**	cag pathogenicity island protein (cag7)	JHP0476	HP0527	**2**	**2**
**3**	type I restriction enzyme (hsdS)	JHP1422	NAH	319	**10**
**4**	vacuolating cytotoxin (vacA) paralog	JHP0556	HP0609/10	**3**	**5**
**5**	vacuolating cytotoxin (vacA) paralog	JHP0274	HP0289	**4**	**6**
**6**	vacuolating cytotoxin (vacA) paralog	JHP0856	HP0922	**7**	**9**
**7**	hypothetical protein	JHP0191	HP0205	**53**	**33**
**8**	hypothetical protein	JHP1044	HP1116	**10**	**14**
**9**	hypothetical protein	JHP0928	NAH	**11**	**12**
**10**	hypothetical protein	JHP0305	HP0322	**5**	**8**
**11**	hypothetical protein	JHP0462	HP0513	104	54
**12**	cag island protein (cagA)	JHP0495	HP0547	**17**	**20**
**13**	type III restriciton enzyme (mod)	JHP1411	HP1522	857	104
**14**	hypothetical protein	JHP1070	HP1142	60	**27**
**15**	type II restriction enzyme (methyltransferase)	JHP1409	NAH	**37**	55
**16**	DNA-directed RNA polymerase, beta subunit (rpoB)	JHP1121	HP1198	**50**	**23**
**17**	hypothetical protein	JHP0440	HP0488	**13**	**16**
**18**	multidrug resistance protein (spaB)	JHP0547	HP0600	75	**39**
**19**	hypothetical protein	JHP0110	HP0118	64	**21**
**20**	topoisomerase I (topA 3)	JHP0931	NAH – check	**18**	**18**
**21**	hypothetical protein	JHP0842	HP0906	**42**	**29**
**22**	type I restriction enzyme (hsdR)	JHP1424	HP1402	195	138
23	type III restriction enzyme (res)	JHP1410	HP1521	161	258
24	outer membrane protein (omp26)	JHP1084	HP1157	**24**	**17**
25	hypothetical protein	JHP1285	HP1371	55	58
26	hypothetical protein	JHP0540	NAH	674	158
27	hypothetical protein	JHP0942	HP0996	**6**	**13**
28	type III restriction enzyme (res)	JHP1297	NAH	**30**	63
29	type I restriction enzyme R protein (hsdR)	JHP0416	HP0464	63	105
30	type I restriction enzyme (hsdS)	JHP0414	NAH	275	85
31	cag pathogenicity island protein (cag8)	JHP0477	HP0528	72	62
32	hypothetical protein	JHP0668	HP0731	110	64
33	hypothetical protein	JHP0613	HP0669	**44**	**42**
34	type III restriction enzyme (mod)	JHP1296	NAH	121	190
35	type I restriction enzyme R protein (hsdR)	JHP0784	HP0846	244	48
36	N-methylhydantoinase	JHP0632	HP0696	**20**	**44**
37	RNA polymerase sigma-70 factor (rpoD)	JHP0081	HP0088	62	71
38	vacuolating cytotoxin (vacA)	JHP0819	HP0887	59	46
39	hypothetical protein	JHP1113	HP1187	274	**28**
40	hypothetical protein	JHP0534	HP0586	577	230
41	type II restriction enzyme (methyltrasferase)	JHP1284	NAH	750	290
42	translation initiation factor IF-2 (infB)	JHP0377	HP1048	330	281
43	restriction enzyme	JHP0164	NAH	88	47
44	hypothetical protein	JHP0458	HP0508	84	50
45	role in outermembrane permeability (imp)	JHP1138	HP1215/6	208	196
46	DNA polymerase I (polA)	JHP1363	HP1470	**46**	81
47	iron(III) dicitrate transport protein (fecA)	JHP0626	HP0686	**38**	89
48	adenine specific DNA methyltransferase (mod)	JHP0244	HP0260	236	122
49	histidine and glutamine-rich metal-binding protein	JHP1321	HP1432	**15**	**4**
50	DNA transfer protein (cagE)	JHP0492	HP0544	**49**	95
					
54	hypothetical protein	JHP0336	HP1089	**27**	51
62	cag pathogenicity island protein (cag3)	JHP0471	HP0522	**21**	**35**
64	hypothetical protein	JHP0026	HP0030	**23**	**36**
70	urease beta subunit (urea amidohydrolase) (ureB)	JHP0067	HP0072	**32**	**37**
82	DNA mismatch repair protein (mutS)	JHP0565	HP0621	**45**	227
84	hypothetical protein	JHP0050	HP0058	**8**	**7**
90	hypothetical protein	JHP0174	HP0187/8/6	**29**	88
95	hypothetical protein	JHP0934	NAH	**16**	**15**
111	iron(III) dicitrate transport protein (fecA)	JHP1426	HP1400	**28**	78
120	hypothetical protein	JHP0052	HP0059	**26**	**24**
125	hypothetical protein	JHP0074	HP0080	**12**	**32**
145	Outer membrane protein	JHP0438	HP0486	**22**	67
154	flagellin A (flaA)	JHP0548	HP0601	**34**	**40**
160	DNA repair protein (recN)	JHP1434	HP1393	**51**	177
209	cytochrome oxidase (cbb3 type) (fixN)	JHP0132	HP0144	**41**	206
275	hypothetical protein	JHP0299	HP061/2	**35**	**11**
342	hypothetical protein	JHP1033	HP1106	**36**	262
384	hypothetical protein	JHP1253	HP1333	**40**	75
393	hypothetical protein	JHP0940	NAH	**39**	53
398	cag island protein (cagH)	JHP0489	HP0541	**47**	137
402	siderophore-mediated iron transport protein (tonB)	JHP1260	HP1341	**48**	1260
541	integrase/recombinase (xerD)	JHP0941	HP0995	**33**	100
558	cag island protein (cagT)	JHP0481	HP0532	**25**	70
694	hypothetical protein	JHP1042	HP1115	**14**	**25**
828	cag island protein (cagF)	JHP0491	HP0543	**52**	170
990	hypothetical protein	JHP0925	NAH	**43**	130
1355	hypothetical protein	JHP0952	HP0427	**1**	**3**
1463	hypothetical protein	JHP0953	NAH	**31**	**26**
1490	hypothetical protein	JHP0693	HP0756	**19**	59

Genes with > 2 SD divergence in each analysis are indicated in **bold**

NAH indicates No Annotated Homologue in the other sequence

**Table 5:** Top 50 most divergent genes by TNS in *H. pylori *strain 26695 plus those additional genes > 2 SD greater than the mean by DNS and the 50 most divergent by HNS

**TNS order**	**annotation**	**HP#**	**J99 #**	**DNS order**	**HNS order**
**1**	cag pathogenicity island protein (cag7)	HP0527	JHP0476	**1**	**1**
**2**	vacuolating cytotoxin (vacA) paralog	HP0289	JHP0274	**2**	**4**
**3**	hypothetical protein	HP0427	JHP0952	**14**	737
**4**	hypothetical protein	HP1408	JHP1300	**15**	738
**5**	vacuolating cytotoxin (vacA) paralog	HP0922	JHP0856	**6**	**3**
**6**	hypothetical protein	HP0609	JHP0556*	**4**	**9**
**7**	hypothetical protein	HP0119	NAH	**17**	**2**
**8**	poly E-rich hypothetical protein	HP0322	JHP0305	**3**	**5**
**9**	histidine and glutamine-rich metal-binding protein	HP1432	JHP1321	**46**	1432
**10**	hypothetical protein	HP0488	JHP0440	**7**	**12**
**11**	hypothetical protein	HP1116	JHP1044	**8**	**13**
**12**	vacuolating cytotoxin (vacA) paralog	HP0610	JHP0556*	**13**	**17**
**13**	secreted protein involved in flagellar motility	HP1192	JHP1117	410	1256
**14**	hypothetical protein	HP0996	JHP0942	**5**	46
**15**	cag island protein (cagA)	HP0547	JHP0495	**31**	**7**
**16**	hypothetical protein	HP0058	JHP0051	121	53
**17**	outer membrane protein (omp26)	HP1157	JHP1084	**34**	25
**18**	hypothetical protein	HP0080	JHP0074	**9**	122
**19**	hypothetical protein	HP1142	JHP1070	91	**6**
**20**	hypothetical protein	HP1520	NAH	167	33
**21**	hypothetical protein	HP0059	JHP0052	**43**	320
**22**	hypothetical protein	HP0906	JHP0842	**42**	**16**
**23**	DNA-directed RNA polymerase, beta subunit (rpoB)	HP1198	JHP1121	84	19
**24**	hypothetical protein	HP0030	JHP0026	**45**	39
**25**	vacuolating cytotoxin (vacA)	HP0887	JHP0819	**18**	34
**26**	histidine-rich, metal binding polypeptide (hpn)	HP1427	NAH	**39**	1449
**27**	hypothetical protein	HP0118	JHP0110	179	36
**28**	hypothetical protein	HP0513	JHP0462	122	**15**
**29**	hypothetical protein	HP1143	JHP1071	**58**	41
**30**	type III restriction enzyme R protein (res)	HP0592	NAH	**16**	35
**31**	hypothetical protein	HP1187	JHP1113	142	38
**32**	hypothetical protein	HP0508	JHP0458	139	77
**33**	hypothetical protein	HP1115	JHP1042	**20**	866
**34**	hypothetical protein	HP1516	NAH	593	1090
**35**	N-methylhydantoinase	HP0696	JHP0632	**19**	43
**36**	hypothetical protein	HP0489	JHP0441	**10**	582
**37**	hypothetical protein	HP0611	JHP0299	230	1129
**38**	urease beta subunit (urea amidohydrolase) (ureB)	HP0072	JHP0067	**21**	87
**39**	integrase/recombinase (xerD)	HP0995	JHP0941	**25**	448
**40**	flagellin A (flaA)	HP0601	JHP0548	**33**	180
**41**	multidrug resistance protein (spaB)	HP0600	JHP0547	97	30
**42**	type IIS restriction enzyme R and M protein (ECO57IR)	HP1517	NAH	**28**	**14**
**43**	fucosyltransferase	HP0651	JHP0596	**48**	75
**44**	hypothetical protein	HP0120	NAH	283	50
**45**	outer membrane protein (omp3)	HP0079	JHP0073	68	99
**46**	hypothetical protein	HP0345	NAH	249	1338
**47**	outer membrane protein (omp17)	HP0725	JHP0662	209	101
**48**	cag pathogenicity island protein (cag3)	HP0522	JHP0471	**11**	100
**49**	virB4 homolog (virB4)	HP0459	NAH	**53**	28
**50**	cag pathogenicity island protein (cag8)	HP0528	JHP0477	74	27
					
**51**	DNA transfer protein (cagE)	HP0441	JHP0492	**29**	22
**53**	hypothetical protein	HP1333	JHP1253	**40**	296
**55**	RNA polymerase sigma-70 factor (rpoD)	HP0088	JHP0081	**56**	31
58	hypothetical protein	HP0453	NAH	75	**10**
60	hypothetical protein	HP0669	JHP0613	69	42
61	hypothetical protein	HP1003	NAH	**38**	170
64	translation elongation factor EF-Tu (tufB)	HP1205	JHP1128	**49**	166
67	hypothetical protein	HP1089	JHP0336	**12**	59
71	hypothetical protein	HP0756	JHP0693	**24**	1548
72	hypothetical protein	HP0788	JHP0725	**41**	256
73	2',3'-cyclic-nucleotide 2'-phosphodiesterase (cpdB)	HP0104	JHP0096	**54**	68
77	DNA polymerase I (polA)	HP1470	JHP1363	**30**	54
78	hypothetical protein	HP0205	JHP0191	**57**	**8**
80	hypothetical protein	HP0731	JHP0668	132	32
81	hypothetical protein	HP0449	NAH	**51**	449
86	type I restriction enzyme R protein (hsdR)	HP1402	JHP1424	103	21
87	cag pathogenicity island protein (cag12)	HP0532	JHP0481	**23**	693
90	type I restriction enzyme R protein (hsdR)	HP0464	NAH	**36**	26
99	iron(III) dicitrate transport protein (fecA)	HP1400	JHP1426	**32**	129
101	type I restriction enzyme R protein (hsdR)	HP0846	JHP0784	342	37
102	cytochrome oxidase (cbb3 type) (fixN)	HP0144	JHP0132	**27**	168
119	type III restriction enzyme R protein	HP1371	JHP1285	**52**	23
120	adenine/cytosine DNA methyltransferase	HP0054	NAH	109	20
130	hypothetical protein	HP0186	JHP0174	**47**	276
137	DNA mismatch repair protein (MutS)	HP0621	JHP0565	**22**	64
147	outer membrane protein	HP0486	JHP0438	**26**	142
149	DNA topoisomerase I (topA)	HP0440	NAH	63	24
153	hypothetical protein	HP1479	JHP1372	**55**	127
154	DNA repair protein (recN)	HP1393	JHP1434	**35**	207
163	hypothetical protein	HP0586	JHP0534	631	29
164	virulence associated protein homolog (vacB)	HP1248	JHP1169	**50**	160
169	GMP reductase (guaC)	HP0854	JHP0790	**44**	451
176	preprotein translocase subunit (secA)	HP0786	JHP0723	119	49
181	cell division protein (ftsK)	HP1090	JHP0335	**37**	90
207	adenine specific DNA methyltransferase (mod)	HP1522	JHP1411	363	**11**
210	type III restriction enzyme R protein (res)	HP1521	JHP1410	195	18
219	DNA polymerase III alpha-subunit (dnaE)	HP1460	JHP1353	297	47
220	type II restriction enzyme (methyltransferase)	HP0478	JHP0430	1080	40
222	hypothetical protein	HP0733	JHP0670	224	48
225	cag pathogenicity island protein (cag13)	HP0534	JHP0482	**60**	1021
272	hypothetical protein	HP1106	JHP1033	**59**	277
332	translation initiation factor IF-2 (infB)	HP1048	JHP0377	291	45
340	type I restriction enzyme M protein (hsdM)	HP1403	JHP1423	125	44

* probably frame shifted components of the same *vacA *related gene

Genes with > 2 SD divergence in each analysis are indicated in **bold**

NAH indicates No Annotated Homologue in the other sequence

**Table 6:** Top 50 most divergent genes by HNS in *H. pylori *strain 26695 plus those additional genes > 2 SD greater than the mean by DNS and the 50 most divergent by HNS

**HNS order**	**annotation**	**HP#**	**J99 #**	**DNS order**	**TNS order**
**1**	cag pathogenicity island protein (cag7)	HP0527	JHP0476	**1**	**1**
**2**	hypothetical protein	HP0119	NAH	**17**	**7**
**3**	vacuolating cytotoxin (vacA) paralog	HP0922	JHP0856	**6**	**5**
**4**	vacuolating cytotoxin (vacA) paralog	HP0289	JHP0274	**2**	**2**
**5**	poly E-rich hypothetical protein	HP0322	JHP0305	**3**	**8**
**6**	hypothetical protein	HP1142	JHP1070	91	**19**
**7**	cag island protein (cagA)	HP0547	JHP0495	**31**	**15**
**8**	hypothetical protein	HP0205	JHP0191	**57**	78
**9**	hypothetical protein	HP0609	JHP0556*	**4**	**6**
**10**	hypothetical protein	HP0453	NAH	75	58
**11**	adenine specific DNA methyltransferase (mod)	HP1522	JHP1411	363	207
**12**	hypothetical protein	HP0488	JHP0440	**7**	**10**
**13**	hypothetical protein	HP1116	JHP1044	**8**	**11**
**14**	type IIS restriction enzyme R and M protein (ECO57IR)	HP1517	NAH	**28**	**42**
**15**	hypothetical protein	HP0513	JHP0462	122	**28**
**16**	hypothetical protein	HP0906	JHP0842	**42**	**22**
**17**	vacuolating cytotoxin (vacA) paralog	HP0610	JHP0556*	**13**	**12**
18	type III restriction enzyme R protein (res)	HP1521	JHP1410	195	210
19	DNA-directed RNA polymerase, beta subunit (rpoB)	HP1198	JHP1121	84	**23**
20	adenine/cytosine DNA methyltransferase	HP0054	NAH	109	120
21	type I restriction enzyme R protein (hsdR)	HP1402	JHP1424	103	86
22	DNA transfer protein (cagE)	HP0441	JHP0492	**29**	**51**
23	type III restriction enzyme R protein	HP1371	JHP1285	**52**	119
24	DNA topoisomerase I (topA)	HP0440	NAH	63	149
25	outer membrane protein (omp26)	HP1157	JHP1084	**34**	**27**
26	type I restriction enzyme R protein (hsdR)	HP0464	NAH	**36**	90
27	cag pathogenicity island protein (cag8)	HP0528	JHP0477	74	**50**
28	virB4 homolog (virB4)	HP0459	NAH	**53**	**49**
29	hypothetical protein	HP0586	JHP0534	631	163
30	multidrug resistance protein (spaB)	HP0600	JHP0547	97	**41**
31	RNA polymerase sigma-70 factor (rpoD)	HP0088	JHP0081	**56**	**55**
32	hypothetical protein	HP0731	JHP0668	132	80
33	hypothetical protein	HP1520	NAH	167	**20**
34	vacuolating cytotoxin	HP0887	JHP0819	**18**	**25**
35	type III restriction enzyme R protein (res)	HP0592	NAH	**16**	**30**
36	hypothetical protein	HP0118	JHP0110	179	**27**
37	type I restriction enzyme R protein (hsdR)	HP0846	JHP0784	342	101
38	hypothetical protein	HP1187	JHP1113	142	**31**
39	hypothetical protein	HP0030	JHP0026	**45**	**24**
40	HP0478	JHP0430	1080	220	
41	hypothetical protein	HP1143	JHP1071	**58**	**29**
42	hypothetical protein	HP0669	JHP0613	69	60
43	N-methylhydantoinase	HP0696	JHP0632	**19**	**35**
44	type I restriction enzyme M protein (hsdM)	HP1403	JHP1423	125	340
45	translation initiation factor IF-2 (infB)	HP1048	JHP0377	291	332
46	hypothetical protein	HP0996	JHP0942	**5**	**14**
47	DNA polymerase III alpha-subunit (dnaE)	HP1460	JHP1353	297	219
48	hypothetical protein	HP0733	JHP0670	224	222
49	preprotein translocase subunit (secA)	HP0786	JHP0723	119	176
50	hypothetical protein	HP0120	NAH	283	**44**
					
53	hypothetical protein	HP0058	JHP0051	121	**16**
54	DNA polymerase I (polA)	HP1470	JHP1363	**30**	77
59	hypothetical protein	HP1089	JHP0336	**12**	67
64	DNA mismatch repair protein (MutS)	HP0621	JHP0565	**22**	137
68	2',3'-cyclic-nucleotide 2'-phosphodiesterase (cpdB)	HP0104	JHP0096	**54**	73
75	fucosyltransferase	HP0651	JHP0596	**48**	**43**
77	hypothetical protein	HP0508	JHP0458	139	**32**
87	urease beta subunit (urea amidohydrolase) (ureB)	HP0072	JHP0067	**21**	**38**
90	cell division protein (ftsK)	HP1090	JHP0335	**37**	181
99	outer membrane protein (omp3)	HP0079	JHP0073	68	**45**
100	cag pathogenicity island protein (cag3)	HP0522	JHP0471	**11**	**48**
101	outer membrane protein (omp17)	HP0725	JHP0662	209	**47**
122	hypothetical protein	HP0080	JHP0074	**9**	**18**
127	hypothetical protein	HP1479	JHP1372	**55**	153
129	iron(III) dicitrate transport protein (fecA)	HP1400	JHP1426	**32**	99
142	outer membrane protein	HP0486	JHP0438	**26**	147
160	virulence associated protein homolog (vacB)	HP1248	JHP1169	**50**	164
166	translation elongation factor EF-Tu (tufB)	HP1205	JHP1128	**49**	64
168	cytochrome oxidase (cbb3 type) (fixN)	HP0144	JHP0132	**27**	102
170	hypothetical protein	HP1003	NAH	**38**	61
180	flagellin A (flaA)	HP0601	JHP0548	**33**	**40**
207	DNA repair protein (recN)	HP1393	JHP1434	**35**	154
256	hypothetical protein	HP0788	JHP0725	**41**	72
276	hypothetical protein	HP0186	JHP0174	**47**	130
277	hypothetical protein	HP1106	JHP1033	**59**	272
296	hypothetical protein	HP1333	JHP1253	**40**	**53**
320	hypothetical protein	HP0059	JHP0052	**43**	**21**
448	integrase/recombinase (xerD)	HP0995	JHP0941	**25**	**39**
449	hypothetical protein	HP0449	NAH	**51**	81
451	GMP reductase (guaC)	HP0854	JHP0790	**44**	169
582	hypothetical protein	HP0489	JHP0441	**10**	**36**
693	cag island protein (cagT)	HP0532	JHP0481	**23**	87
737	hypothetical protein	HP0427	JHP0952	**14**	**3**
738	hypothetical protein	HP1408	JHP1300	**15**	**4**
866	hypothetical protein	HP1115	JHP1042	**20**	**33**
1021	cag pathogenicity island protein (cag13)	HP0534	JHP0482	**60**	225
1090	hypothetical protein	HP1516	NAH	593	**34**
1129	hypothetical protein	HP0611	JHP0299	230	**37**
1256	secreted protein involved in flagellar motility	HP1192	JHP1117	410	**13**
1338	hypothetical protein	HP0345	NAH	249	**46**
1432	histidine and glutamine-rich metal-binding protein	HP1432	JHP1321	**46**	**9**
1449	histidine-rich, metal binding polypeptide (hpn)	HP1427	NAH	**39**	**26**
1548	hypothetical protein	HP0756	JHP0693	**24**	71

* probably frame shifted components of the same *vacA *related gene

Genes with > 2 SD divergence in each analysis are indicated in **bold**

NAH indicates No Annotated Homologue in the other sequence

The 50 most divergent J99 ORFs by HNS included 26 (52%) that were not in the 53 (>2 SD) most divergent by DNS, these included 11 restriction-modification system genes and 6 others that were not annotated within the strain 26695 genome sequence. The identification of genes of a type known to be horizontally exchanged, and different between the gene complements of the strains, is strong corroboration for the foreign origin of the additional genes identified by HNS. In several instances (Tables 2 to 6) the DNS did not detect these genes at all e.g. restriction enzymes that were the 3^rd^, 13^th ^and 41^st ^most divergent genes by HNS, were 319^th^, 857^th ^and 750^th ^most divergent by DNS, respectively. In some instances the TNS gave intermediate results and in others identified other genes as more divergent than the other methods. The TNS was most sensitive for the detection of *rpoB *(HP1198 / JHP1121) which is associated with a significantly different gene length in the two strains (Figure 1h). One explanation for this observation is that while the DNS may initially be the most sensitive indicator of horizontal exchange it may become ameliorated to the new sequence characteristics more rapidly that the longer component features, which are probably detecting qualitatively different sequence characteristics.

The differences in the analyses using different length components, and a comparison of the results from the two sequenced strains, suggest a complex evolutionary history for the *cag *pathogenicity island. These suggest that it probably has mosaic structure including sequences from more than one species background, in addition to sequence that is entirely typical of *H. pylori*.

It is normally impossible to determine the chronology of events to distinguish insertions and deletions when comparing strains. In strain 26695 there are two open reading frames that are both good candidate coding sequences. There is only one gene in this location in strain J99 composed of the 5' gene from strain 26695 and the 3' end of the subsequent gene. This could have arisen from either a deletion or an insertion event. However, the normal DNS of the J99 gene (JHP0073, 799^th ^in divergence) and the 5' 26695 gene (HP0079, 751^st ^in divergence), and the high divergence of the 3' 26695 gene (HP0078, 68^th ^in divergence), indicate that the most likely event is an insertion into strain 26695 (Figure 1l). Likewise HP0119 is likely to contain an insertion and JHP1113 probably reflects the original sequences (Figure 1k).

The inclusion of two DNA metabolism genes associated with recombination and repair is notable. Both *mutS *and *recN *were identified in both strains (22^nd ^and 35^th^, and 45^th ^and 51^st ^most divergent genes by DNS in strains 26695 and J99 respectively). When the homologous genes were compared between the strains, extensive divergences were evident between more than one region of each protein. That these genes have divergent signatures in both strains suggests that neither has a wholly native composition. This observation is consistent with the models of rapid evolution which suggest that transient competitive advantages are enjoyed by organisms that are hypermutators under conditions of environmental stress and transitions, and that these states which can be produced by mutations in DNA repair genes [21-26]. However, such states have to be reversed so that an unsustainable mutational burden is not attained, and it has been proposed that this reversal is mediated by repair following horizontal transfer and homologous recombination, and that such strains are hyper-recombinogenic [27-29]. The untypicality of *mutS *and *recN *suggest that *H. pylori *is another species that can make use of this strategy for diversification under stressful conditions.

The identification of RNA polymerase genes, with associated differences between the strains, is striking. The divergence of phylogenetic trees based upon different sequences has been highlighted, and particularly the differences between the trees associated with RNA polymerase genes and rRNA [30,31]. It has been argued that RNA polymerase is as essential to cell function as is rRNA and that there is no compelling reason to chose rRNA as the more reliable marker [32]. While the DNS analysis does not address the stability of rRNA (and specifically excludes the rRNA sequences because their differing coding requirements and evolutionary pressures generate a divergent signature for other reasons), it does indicate that RNA polymerase can be a substrate for horizontal transfer, and that trees based upon this gene, or other essential genes, need not necessarily be considered a challenge to rRNA based phylogenies.

## Conclusions

The spectrum of recently horizontally acquired sequences identified emphasizes the two driving forces of horizontal exchange: the transfer of a phenotype which alters or enhances bacterial fitness resulting in increased competitive fitness or altered niche adaptation, and the presence of a substrate for homologous recombination. Because of the focus upon, and relative ease of identifying, large islands associated with readily identifiable features and phenotypes, the importance of the latter component has perhaps been underestimated. The genes that have been considered to code for 'core metabolic' 'house-keeping' functions are amongst those most likely to be changed by horizontal transfer events because of the presence of homologous substrates, and changes are likely to persist even when the change is phenotypically neutral. Equally, changes in the genes involved in core functions such as gene expression and DNA metabolism may have pleotropic effects and there may be significant differences in strain behaviour, that are not simply the consequence of differences in their respective gene complements. The selection of genes for phylogenetic analysis on the basis of their coding for conserved core functions is also problematic because these are also frequently the genes most likely to share the high homology that facilitates recombination and horizontal exchange.

## Methods

A traditional nucleotide signature is generated by segmenting a sequence of DNA into *k *equal-sized subsequences (or 'windows'). The mathematical basis for the signature is an odds ratio – *p*_*i *_– calculated by dividing the frequency of a length-*L *oligonucleotide by its expected frequency. The odds ratios for each of the 4^*L *^oligonucleotides in each window (*w*) are compared with the odds ratios for the overall sequence (*s*) [9,10,33]. The normalized difference *δ *is plotted and thus a nucleotide signature consists of a *k*-length sequence of *δ *values: *δ*(*w*,*s*) = (1/4^*L*^)Σ(4^*L*^,*i*:*x*)|*p*_*i*_(*w*) - *p*_*i*_(*s*)|, where *x *is the set of all permutations of length *L *and *i *is one such permutation.

There are interesting parallels between signature-style genome analysis and stylometric techniques previously used to determine the authorship of controversial literary texts. This is analogous with the biological problem and it is from this that our method is derived. Rather than using a fixed-window signature, signature scores are calculated for each coding open reading frame (ORF) and weighted with variance estimates so that the scores for shorter ORFs confer with their longer counterparts. Bissell's *weighted cusum *(cumulative sum) [34], , is modified so that *n *denotes the number of ORFs in the genome, *X*_*i *_the number of oligonucleotides in ORF *i*, and *w*_*i *_the number of nucleotides in ORF *i*. The results are scaled according to ORF size using the standard error *σ *= √(*#*ORF*). In this way false positives are abrogated by normalizing for over-representation of lower order peptides.

The method is implemented in Java and efficiency is maintained through an *O*(*N*) (*N *= sequence length) refinement: probabilities for the complete sequence are calculated in *O*(*N*) steps for any length-*L *oligonucleotide, and maintain *O*(*N*) when *4*^*L*^>*N *through a hashing function; the second part of the program calculates *σ *for each ORF using a loop flattening technique, thereby avoiding the program having to recalculate overlapping sub-expressions. The program is available from  and .

Sequence alignments, as shown in Figure 1, were performed and displayed using the programs: Lalign and viewed using Lalignview [35].

## Abbreviations

ORF, Open Reading Frame; DNS, Dinucleotide Signature; TNS, tetranucleotide signature; HNS, hexanucleotide signature.

## Authors' contributions

NJS initiated the project, performed the genome sequence analyses, compared the two strains, interpreted the results, and prepared the biological aspects of the manuscript. PB was a DPhil student who worked on the coding aspects of the new methodology. JFP contributed to the bioinformatics discussions and planning stage of this project. SAJ directed and primarily developed the analysis strategy and the implementation of the new computational basis of the methodology, and prepared the computational aspects of the manuscript.
